# Reliability of London atlas for dental age estimation in an Australian cohort

**DOI:** 10.1007/s00414-025-03559-2

**Published:** 2025-07-09

**Authors:** Amanda Chua, Christabel Halim, Ethan Pham, Selwin Samuel, Sobia Zafar, Sakher AlQahtani

**Affiliations:** 1 School of Dentistry, The University of Queensland, Brisbane, Australia; 2 Department of Oral Pathology and Microbiology, Saveetha Institute of Medical and Technical Sciences (SIMATS), Chennai, Tiruvallur India; 3 Department of Pediatric Dentistry and Orthodontics, College of Dentistry, King Saud University, Riyadh, Saudi Arabia; 4 School of Dentistry, The University of Queensland, 288 Herston Rd, Herston, QLD 4006 Australia

**Keywords:** London atlas, Dental age estimation, Australian population, Panoramic radiographs

## Abstract

**Introduction:**

Age estimation is a very essential tool that is required for quite a lot of purposes in legal settings and for disaster victim identification purposes. In the recent years, London Atlas for dental age estimation has gained popularity and its applicability in Australia has not been sufficiently validated. Therefore, a study was aimed to study the ability of London Atlas to accurately estimate the ages of an Australian cohort.

**Aim:**

To test the age prediction reliability of the London Atlas on an Australian population and to determine if there is a difference in its prediction accuracy between males and females.

**Methods:**

A total of 193 panoramic radiographs were accessed from the University of Queensland’s archival records. The London Atlas was used to estimate the dental ages of these radiographs of 96 females and 97 males, aged between 5 and 17 years.

**Results:**

Of the entire cohort, the difference between the mean estimated age (11.56 years) and mean chronological (11.92 years) age was 0.36 years. This difference was found to be statistically significant (*p* < 0.001). The over-estimation of ages was significant in age groups 6, 7, 8,10 and 11. The mean age difference for males was − 0.038 years while the difference for females was 0.471 years. However, the difference between the two sexes (0.509 years) was statistically insignificant (*p* > 0.001). The London Atlas shows a tendency to over-estimate ages of females and under-estimate ages of males.

**Conclusion:**

The London Atlas was found to overestimate the ages of children in an Australian population by approximately four and a half months (0.37 years). However, there was no difference in age prediction accuracy between males and females. Overall, the London Atlas has comparable accuracy with other dental age estimation methods and should be considered as a tool for age estimation.

**Supplementary Information:**

The online version contains supplementary material available at 10.1007/s00414-025-03559-2.

## Introduction

The age of an individual is a critical characteristic that has far-reaching implications on the society [1]. Age determines a person’s eligibility to attend school, seek work, and participate in various social activities [1, 2]. It also determines how the justice system handles cases of child labour, child abuse, child marriage, adoption, kidnapping and criminal cases involving minors [2, 3]. Age estimation is necessary as it helps to identify unknown or missing persons; identify casualties in large-scale disasters; expedite the process of determining the age of illegal migrants or migrants lacking valid documentation [2]. The medico legal consequences of unidentified individuals are dire, hence, methods for age estimation are crucial to assess the age of individuals in such cases [2].

Several methods have been used for age estimation, such as radiological examination of dental development, radiological examination of skeletal development and aspartic acid racemization in dentine [4–7], to name a few. It has been shown that tooth development and eruption patterns are more consistent with the age of individuals as they are less prone to being affected by environmental and nutritional factors [5, 6]. Additionally, while some studies show that age estimation using aspartic acid racemization in dentine produce more precise results, this method requires extracted teeth which is not ideal in the case of living individuals [6]. Hence, assessing dental age using radiographs and a standardized tooth development chart is routinely practised in academic and clinical settings, and has the potential to be the most practical and reliable method as it is non-invasive and more practical in living individuals.

Tooth development stages can be assessed via tooth specific methods or through dental charts [5]. Tooth specific methods predict age from a specific developmental stage of a single tooth, which can be influenced by the age distribution and age range of the test sample. Therefore, an analysis by age categories via dental charts which cover the whole dentition, can provide a more reliable information.

One of the oldest and well-known dental age estimation charts is the atlas of Schour and Massler (1941), which consist of 21 drawings of human dentition between the age of five months and 35 years [8]. However, the lack of drawings for ages 12 to 15 and 15 to 21, gives unreliable results for older subjects. Ubelaker’s dental chart (1978) was an attempt to improve the atlas of Schour and Massler (1941) [8, 9]. Ubelaker utilised published sources to correct the age range for each drawing and covered more variations for each age range [10]. This chart has also been modified for use in Australia, with separate charts for males and females [10]. Several other similar charts were also developed as a result, such as Gustafson and Koch (1974), Brown (1985), Kahl and Schwarze (1988) [5]. However, none of these charts proved more superior than the other [5].

Other proven radiographic age estimation techniques involve a scoring system such as Demirjian’s and Willem’s methods [11, 12]. These methods involve assignment of scores to the seven left mandibular permanent teeth (excluding third molars). The Demirjian’s method calculates a dental maturity score by summing the assigned scores for each tooth, while the Willem’s method adapts this approach to directly convert the total score into an estimated dental age in years [13].

The London Atlas was developed as an evidence-based atlas to assess dentition for age estimation. It is comprehensive, consisting of 31 age categories, using both tooth development and alveolar eruption of individuals [5]. Tooth development and eruption is illustrated in this atlas for ages 1 to 23, with diagrams at the midpoint of each chronological year [5]. “The London Atlas” has emerged as one of the more accurate and reliable methods of age estimation in the British Bangladeshi and Hispanic population [4, 5]. However, this has not yet been tested in an Australian population. This study will test the accuracy and reliability of “The London Atlas” as an age estimation tool in an Australian population.

## Aims and hypotheses

The aim of this study is to test the reliability of The London Atlas in an Australian population. Two hypotheses will be tested:

i. there will be a difference between the chronological age and the age predicted by the London Atlas,
ii. there will be a difference in the prediction accuracy of the London Atlas between males and females.

## Materials and methods

This was a retrospective cross-sectional study of orthopantomograms (OPG) of individuals between the ages 5 and 17 years. Dental records of patients, who consented for their information to be used for research purposes, were included in this study.

### Ethics approval

Ethics approval for this study was reviewed by Human Research Ethics Committee at The University of Queensland (UQ) via the low-risk review pathway. The risk of breach of privacy was controlled via de-identification and minimized as only examiners (S.Z and S.A) had access to the identified records. 

### Sample selection

The total number of radiographs obtained from the university’s archives was 600. This sample size (*n* = 193) was calculated using a single mean estimation with standard deviation (SD) of 14 units in maturity score and precision of 5 units.

### Selection process

Good quality OPGs were selected (*n* = 193) with all teeth in focus, of healthy individuals aged 5–17 years from the archival records of the University of Queensland (UQ), School of Dentistry. Of the 193 radiographs, 49.7% were male and 50.3% were female. Excluded samples comprised of unclear radiographs, patients with hypodontia, hyperdontia, gross anomalies and pathologies (e.g. Taurodontism, microdontia, Amelogenesis Imperfecta, Dentinogenesis Imperfecta, tumours, abscesses, cysts, fractures etc.), presence of gross caries, previous orthodontic treatment or severe malocclusion.

### De-identification of radiographs

**Fig. 1:**
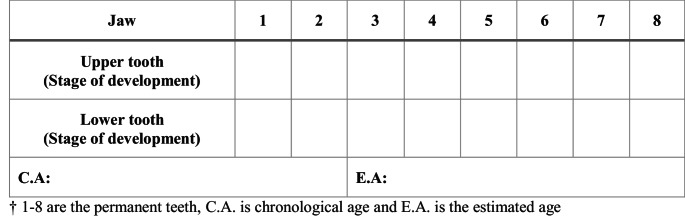
Results table: Estimated stage of tooth development and age

The dental record archives were reviewed by a paediatric dentist and a forensic odontologist (S.Z and S.A). Radiographs were selected based on the inclusion and exclusion criteria of the study as given in Fig. 1. Subjects who satisfied the criteria had their gender, date of birth and date of the radiograph collected. This data was de-identified and assigned a study identification number to maintain patient confidentiality. This study identification number is generated by a random number generator (RANDOM.ORG). The examiners maintained a separate link between the study identification and the patient’s electronic health record number. The de-identified radiographs were assigned to three assessors who were fifth year dental students at UQ.

### Age Estimation using the London atlas

**Fig. 2:**
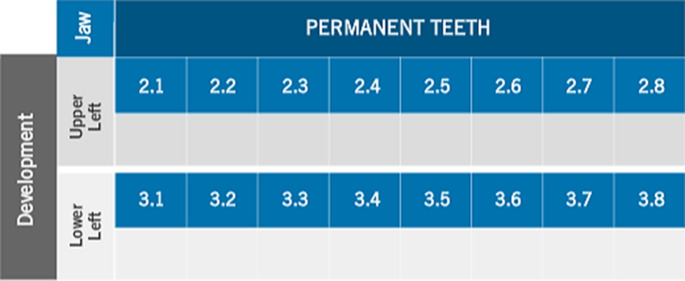
Software entry table: tooth developmental stage

The de-identified radiographs were assessed by the UQ dental students (*n* = 3) to determine the developmental and eruption stages of all teeth on the left side, in both upper and lower jaws, according to the London atlas [12]. Stage of development and eruption of each tooth was entered in the template table (Fig. 1). The estimated age was generated using the Lond Atlas Software (Queen Mary Innovation Ltd, London, United Kingdom) (Fig. 2).

The chronological age for each subject will be calculated by subtracting the date of the radiograph from the date of birth after having converted both to a decimal age using Eveleth and Tanner’s method [11]. The chronological age was blinded from the evaluators until after the age estimation was completed for all radiographs.

### Analysis of data

The chronological age was subtracted from the estimated age, a positive result suggests an overestimation in age while a negative result suggests an underestimation. The mean difference of chronological age and estimated age of the whole sample was calculated. The mean difference for each age category was also calculated. A paired sample t-test was used to determine if the mean difference is significant. The mean difference in chronological age and estimated age for males and females were compared with the independent sample t-test. This will assess the accuracy of the London Atlas in age estimation between males and females. For all analyses, a p-value of less than 0.05 was considered a statically significant difference.

### Inter-examiner and Intra-examiner reliability

**Fig. 3:**
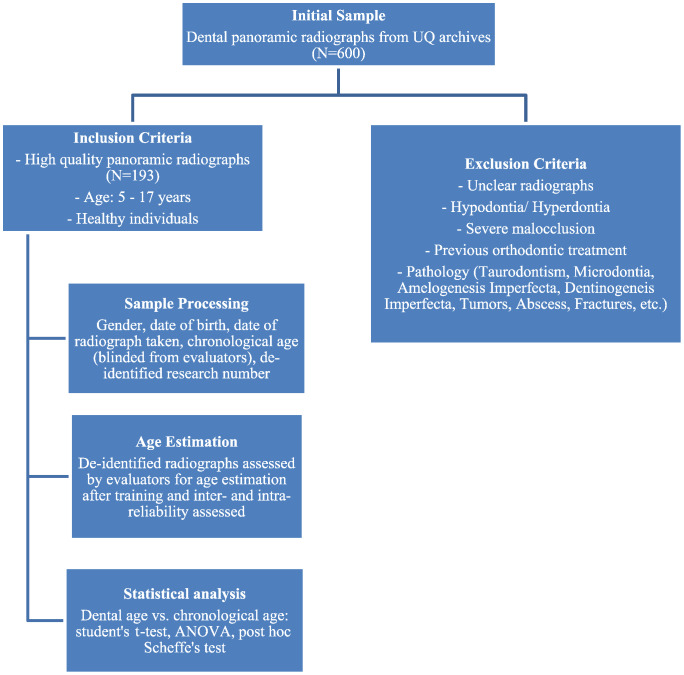
Flow diagram of the methodological approach

Before the data collection process, the three student assessors underwent calibration to ensure consistency and accuracy in their evaluations. Each assessor was first subjected to inter- and intra-examiner reliability testing using a sample of 10 radiographs. Following satisfactory reliability results, all students were provided with the full set of radiographs, and each student independently assessed the images. For inter-examiner reliability, each student’s assessments were compared with those of an experienced examiner (S.A.) using Cohen’s kappa coefficient. To assess intra-examiner reliability, the same sample was re-evaluated by the students after a two-week interval, again using Cohen’s kappa (Fig. 3).

## Results

Of the 600 radiographs, only 193 satisfied the inclusion criteria, where 96 radiographs were of female subjects and 97 radiographs were of male subjects. Pre-data collection, inter-examiner and intra-examiner tests yielded Cohen’s Kappa scores of 0.89 and 0.81 respectively (Table 1).

**Table 1:** Inter and Intra-examiner kappa values

Raters	Inter-examiner reliability	Intra-examiner reliability
Rater 1	0.880	0.840
Rater 2	0.900	0.800
Rater 3	0.890	0.800

The mean estimated age for the collected sample was 11.56 years whilst the mean chronological age was 11.92 years. The mean difference of + 0.36 (standard deviation = 1.67) was found to be significant (*p* = 0.003). The overestimation of ages in age groups 6,7, 8, 10 and 11 were statistically significant, but were insignificant in age groups 9, 12, 13 and 15. The two largest overestimations (1.11 years and 1.05 years respectively) were found in age group 10 and 11.The atlas underestimated ages in groups 14 and 16, with the largest underestimation of 0.98 years in the 14-14.99 years age group, but the mean difference was statistically insignificant. Overall, the London Atlas was found to significantly over-estimate ages of the Australian children (Table 2).

**Table 2:** Mean difference between chronological and estimated age by age groups

Age group (years)	No.	Mean difference	SD*	Sig. (*p* < 0.05)	SE*	95% confidence interval of the difference		t-score
						Lower	Upper	
All samples	193	0.362	1.672	0.003	0.122	0.119	0.608	2.990
6-6.99	11	0.578	0.553	0.006	0.162	0.206	0.949	3.461
7-7.99	19	0.308	0.615	0.043	0.135	0.114	0.604	2.182
8-8.99	21	0.716	1.022	0.004	0.224	0.250	1.181	3.209
9-9.99	20	0.339	1.437	0.305	0.308	−0.334	1.011	1.054
10-10.99	12	1.112	1.149	0.006	0.325	0.382	1.842	3.352
11-11.99	20	1.050	1.962	0.027	0.426	0.132	1.968	2.393
12-12.99	19	0.039	1.391	0.796	0.307	−0.587	0.754	0.263
13-13.99	17	0.685	2.004	0.178	0.473	−0.346	1.715	1.409
14-14.99	18	−0.980	2.132	0.848	0.488	−1.158	0.962	−0.195
15-15.99	19	0.107	2.457	0.852	0.544	−1.077	1.292	0.190
16-16.99	15	−0.842	1.675	0.072	0.412	−1.770	0.853	−1.948

*SD – Standard deviation; SE – Standard error

Table 3 summarises the mean age difference (difference between chronological and estimated age) in males and females. The London Atlas underestimated the ages of males by 0.038 years and overestimated the ages of females by 0.471 years.

**Table 3:** Estimated and chronological age difference in males and females (years)

Sex	Females	Males
Mean difference	0.471	−0.038
Standard deviation	2.560	1.772

The results for males and females were compared using an independent samples t-test (Table 4). The age difference between males and females is 0.509. However, the difference in variance and difference between the two genders were not significant (*p* = 0.575 and *p* = 0.110 respectively). Therefore, there was no significant difference in the accuracy of the London Atlas in estimating ages of males or females.

**Table 4:** Difference between estimated and chronological ages of male and female subjects

Independent samples test		Levene’s test for equality of variances		t-test for equality of means						
		F*	sig*	t*	df*	Sig*	Mean diff	SE diff*	95% CI*	
									Lower	Upper
Difference: males vs females	Equal variances assumed	0.316	0.575	1.607	191.000	0.110	0.509	0.317	−0.116	1.133
	Equal variances not assumed			1.604	168.889	0.111	0.509	0.317	−0.118	1.135

*F – Fisher ratio; sig — significance; t — t-value; df — degrees offreedom; SE diff — standard error difference; 95% CI — 95% confidence interval

## Discussion

Dental development has shown to be one of the more uniform processes in humans and it strongly correlates with the actual (chronological) age of an individual. Dental radiographs are often used to estimate dental age as it can be used in living individuals, is economical, easy to access and reliable [14]. This study investigated the reliability of the London Atlas in age estimation of the Australian population. This was carried out by measuring the difference in chronological and estimated age of the individual, which indicated either an overestimation or underestimation of chronological age.

Using the London Atlas, there was an underestimation in the age for males and overestimation in the age for females. This supports studies using Demirjian’s methods, where the overestimation and underestimation of ages were explained by slight discrepancies in the dental development between males and females [11, 15]. However, there was no significant difference in the accuracy of the London Atlas in estimating ages of males or females, and this coincides with results obtained from the study of the London Atlas in the Hispanic and Thai population [16, 17].

The London Atlas was generally found to overestimate the ages of children in the Australian population. This was an average overestimation of 0.36 years, which equates to approximately four and a half months. This difference of four and a half months of age would not result in significant undesirable consequences, when applied to real world circumstances. The overestimation bias was statistically significant in several of the younger age groups, indicating that the atlas may be more inaccurate and overestimates more so in the younger population of Australian children. This slight overestimation was consistent with the results obtained when the London Atlas was applied to the Thai population [17]. Furthermore, in this study, children aged 10 and 11 had the largest over estimation (1.11 years and 1.05 years respectively). Recent studies on the Hispanic and Brazilian children population gave similar results. When the London Atlas was applied, Hispanic children aged 11 to 12 and Brazilian children aged 11 had one of the largest overestimations [16, 18].

The average overestimation of 0.36 years from this study is consistent with the study conducted in Hispanic children using The London Atlas, where an average overestimation of 0.35 years was obtained [16]. This result also concurs with the findings of the London Atlas in the Portuguese population, although the overestimated amount was one month, which was lesser than that in this study [19]. Additionally, The London Atlas during this study proved similar estimation to Demirjian’s methods previously tested in various Australian populations [11, 12]. Flood et al. used Demirjian’s methods including more recent modified methods and found average age overestimation ranging from 0.31 to 0.61 years in a South Australian population [12]. This method is usually found to be more time consuming due to more in depth analysis of each individual tooth.

This study utilised an almost equal distribution of males (*n* = 97) and females (*n* = 96). Additionally, a significant number of radiographs were initially obtained to ensure a higher probability of members from the population to be included in the dataset. These radiographs were also filtered according to a strict inclusion and exclusion criteria, which produced 193 suitable radiographs.

This study had quite a few limitations. While the radiographs had strict inclusion and exclusion criteria, the quality of the radiographs could have varied. These images were taken on different machines in the facility and taken by different personnel. This non-standardised quality could have affected the interpretation of the developmental stages. Besides, all three intra-examiner reliability scores were consistently slightly lower than that of the inter-examiner scores which can possibly be due to factors such as examiner fatigue or a learning effect that may have influenced the intra-examiner results. While this is a potential limitation, the values were still 0.800 and above and hence it does not detract from the overall reliability and validity of the assessment process used for this study.

The London atlas age estimation method could also be limited by the variation in the development of third molars. However, to this date, it is debatable whether the inclusion of third molars in age estimation affects the accuracy of the results [20]. It is evident that third molars vary in development, morphology and positioning, and could potentially introduce more error in predicting one’s age [20]. Several studies even proposed that the rate of third molars developing, differs across different countries and ethnic groups [20, 21]. However, these studies concluded that the small differences in third molar development had little impact on age estimations due to the large standard deviation of developmental stages in each age group [20, 21]. Additionally, this population-specific study targeting solely the Australian population, reduced the error of possible cross-population variability. The study of the London Atlas for age estimation in Portugese population also concluded that third molars should be used in age estimation as the results yielded are as reliable as using a whole set of teeth [19]. Another limitation of the London atlas is that it estimates the age to the precision of the midpoint of each year (e.g. 6.5 years), whereas other studies including Demirijian’s methods, produces point estimates of age. However, the London Atlas has proven to be superior in accuracy when compared with most point estimate methods [5, 11].

The difference between the dental age and chronological age might also not be attributed to the shortcomings of the respective dental age estimation methods. Similar to other biological processes of the human species, dental development also has a certain amount of variation [14, 16]. Therefore, this difference might not be a limitation of the respective age estimation method but a limitation to using tooth eruption patterns to estimate chronological age [16].

It is likely that technologies like machine learning and artificial intelligence (AI) will play a larger role in forensic age estimation moving forward, as other studies also suggest [22]. However, we believe it is crucial to first grasp the foundational principles of this area of research before relying on advanced technologies, so as to better interpret the outcomes of automated methods. The London Atlas is emerging amongst many other age estimation methods. It is easily accessible in various languages and has comprehensive illustrations of various developmental stages and age categories. The results of the present study are comparable with other studies and also concludes The London Atlas as a reliable tool for age estimation. The London Atlas will need to be performed in different countries or ethnic groups to assess its robustness in age estimation across populations. One way of doing this would be to do a targeted study on Australian Aboriginals and Torres Strait Islanders versus Caucasoids. Reports have shown that Australian Aboriginals and Torres Strait Islanders have faster dental development than those of European descent [23]. A comparison of the London Atlas and another age estimation method on the specific target group would be beneficial in determining which method is more accurate. Furthermore, obtaining a large enough sample was a difficulty this study faced. This could be made possible in the future by utilising radiographs from multiple Queensland Health facilities.

## Conclusion

Age estimation has significant relevance in current society. It guides decisions regarding child rights, justice, medico-legal disputes, migration and forensic science. The London Atlas is an emerging age estimation tool that is easily accessible, reproducible when used, and comprehensible. In this study, The London Atlas generally marginally overestimated the ages of Australian children and is equally accurate in estimating the age of males and females. Nevertheless, all current age estimation methods have shown its shortcomings. Overall, the London Atlas has comparable accuracy with other dental age estimation methods and should be considered as an adjunct tool for age estimation in Australia.

## Electronic supplementary material

Below is the link to the electronic supplementary material.

Supplementary Material 1 (DOCX 767 KB)

## Data Availability

All data generated or analysed during this study are included in the manuscript or are available from the corresponding author upon reasonable request.
