# Supplementary material for: Reliability of London atlas for dental age estimation in an Australian cohort
Source: Int J Legal Med. 2025 Jul 9;139(6):2913–20. doi: 10.1007/s00414-025-03559-2 (PMC12532637; doi:10.1007/s00414-025-03559-2)

**SUPPLEMENTARY DATA**

***Supplementary Figure 1.*** The London Atlas: Atlas of human tooth development and eruption (4).


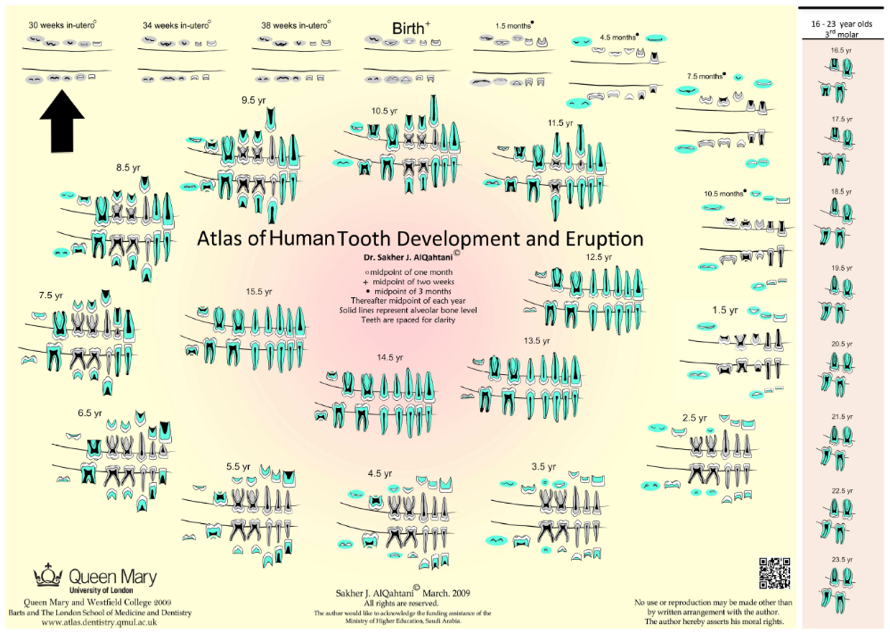


***Supplementary Figure 2.*** Individual tooth development stages, adapted from Moorrees’ Stages (24)


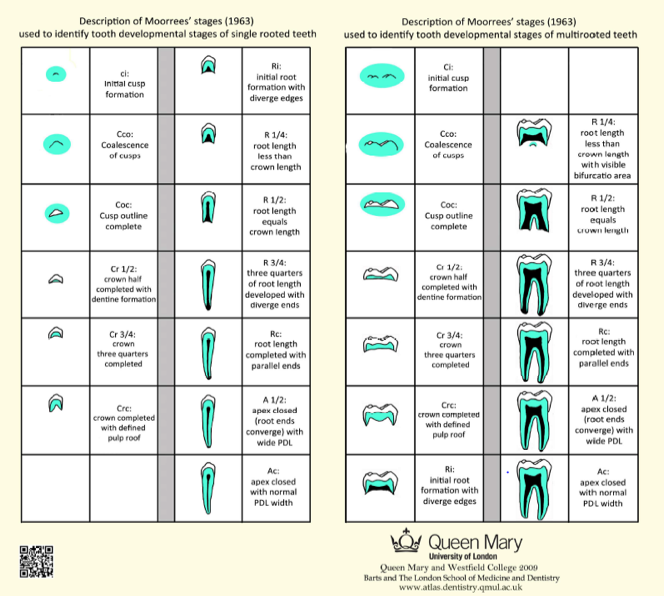

Supplement: Supplementary file 1 — Supplementary Material 1 (DOCX 767 KB) [file 414_2025_3559_MOESM1_ESM.docx]
